# Mechanisms Involved in the Nociception Triggered by the Venom of the Armed Spider *Phoneutria nigriventer*

**DOI:** 10.1371/journal.pntd.0002198

**Published:** 2013-04-25

**Authors:** Camila Gewehr, Sara Marchesan Oliveira, Mateus Fortes Rossato, Gabriela Trevisan, Gerusa Duarte Dalmolin, Flávia Karine Rigo, Célio José de Castro Júnior, Marta Nascimento Cordeiro, Juliano Ferreira, Marcus V. Gomez

**Affiliations:** 1 Programa de Pós-graduação em Ciências da Saúde: Biomedicina e Medicina, Instituto de Ensino e Pesquisa da Santa Casa de Belo Horizonte, Grupo Santa Casa de Belo Horizonte, Belo Horizonte, Minas Gerais, Brazil; 2 Programa de Pós-graduação em Ciências Biológicas: Bioquímica Toxicológica, Universidade Federal de Santa Maria, Santa Maria, Rio Grande do Sul, Brazil; 3 Fundação Ezequiel Dias, Belo Horizonte, Minas Gerais, Brazil; King's College London, United Kingdom

## Abstract

**Background:**

The frequency of accidental spider bites in Brazil is growing, and poisoning due to bites from the spider genus *Phoneutria nigriventer* is the second most frequent source of such accidents. Intense local pain is the major symptom reported after bites of *P. nigriventer*, although the mechanisms involved are still poorly understood. Therefore, the aim of this study was to identify the mechanisms involved in nociception triggered by the venom of *Phoneutria nigriventer* (PNV).

**Methodology/Principal Findings:**

Twenty microliters of PNV or PBS was injected into the mouse paw (intraplantar, i.pl.). The time spent licking the injected paw was considered indicative of the level of nociception. I.pl. injection of PNV produced spontaneous nociception, which was reduced by arachnid antivenin (ArAv), local anaesthetics, opioids, acetaminophen and dipyrone, but not indomethacin. Boiling or dialysing the venom reduced the nociception induced by the venom. PNV-induced nociception is not dependent on glutamate or histamine receptors or on mast cell degranulation, but it is mediated by the stimulation of sensory fibres that contain serotonin 4 (5-HT_4_) and vanilloid receptors (TRPV1). We detected a kallikrein-like kinin-generating enzyme activity in tissue treated with PNV, which also contributes to nociception. Inhibition of enzymatic activity or administration of a receptor antagonist for kinin B_2_ was able to inhibit the nociception induced by PNV. PNV nociception was also reduced by the blockade of tetrodotoxin-sensitive Na^+^ channels, acid-sensitive ion channels (ASIC) and TRPV1 receptors.

**Conclusion/Significance:**

Results suggest that both low- and high-molecular-weight toxins of PNV produce spontaneous nociception through direct or indirect action of kinin B_2_, TRPV1, 5-HT_4_ or ASIC receptors and voltage-dependent sodium channels present in sensory neurons but not in mast cells. Understanding the mechanisms involved in nociception caused by PNV are of interest not only for better treating poisoning by *P. nigriventer* but also appreciating the diversity of targets triggered by PNV toxins.

## Author Summary

Spiders of the *Phoneutria* genus live in Central and South America, where relevant envenomation cases have been reported in humans. The incidence of bite by spiders in Brazil has increased in recent years, with *Phoneutria nigriventer* being the second most important cause of such accidents (approximately 4,000 cases of envenomation in 2011). Pain is the primary local symptom of inoculation with *Phoneutria nigriventer* venom (PNV), but the mechanisms involved in pain induced by PNV are poorly understood. It is important to find effective treatments to alleviate this pain. This study examined the mechanisms involved in pain caused by PNV in a mouse model as well as the sensitivity of PNV-induced pain to clinically used analgesics. The results show that both the low- and high-molecular-weight components of PNV produce spontaneous nociception action via kinin B_2_, TRPV1, 5-HT_4_ or ASIC receptors and the voltage-gated Na^+^ channels present in sensory fibres. Moreover, PNV-triggered nociception could be alleviated by arachnid antivenin, local anaesthetics, opioids and atypical, but not typical, non-steroidal anti-inflammatory drugs. The elucidation of the mechanisms responsible for the nociception induced by PNV is of interest to not only better treat envenomation by *P. nigriventer* but also understand the diversity of targets triggered by PNV toxins.

## Introduction

Spiders of the *Phoneutria* genus, popularly known as the wandering or banana spider, are found in Central and South America, where relevant envenomation cases in humans have been reported [1]. There are four main *Phoneutria* species, *P. fera*, *P. reidyi*, *P. keyserlingi* and *P. nigriventer* [2]. The incidence of accidents due to spiders in Brazil has grown in recent years (from 1.9 in 2000 to 13.7 cases per 100,000 habitants in 2011), making *Phoneutria nigriventer* bites the second most important causes of such accidents. Approximately 4,000 cases of envenomation were reported to the Brazilian Ministry of Health in 2011 [Unpublished data. SINAN-Animais Peçonhentos/SVS/MS. http://dtr2004.saude.gov.br/sinanweb/tabnet/dh?sinannet/animaisp/bases/animaisbrnet.def]. Thus, *Phoneutria nigriventer* envenomation is an important public-health problem in Brazil, which is aggravated by the fact that *P. nigriventer* is very aggressive and is frequently found in human dwellings as a result of the accumulation of organic waste, which attracts insects, the natural prey of these spiders [1].

Envenomation by *Phoneutria nigriventer* venom (PNV) in humans may produce systemic symptoms, such as spastic paralysis or tremors, and local symptoms, such as pain and oedema. Intense local pain is the main clinical manifestation following *P. nigriventer* envenomation, appearing in 96% of envenomed patients [3], [1]. The development of local oedema is less frequent than pain and appears in 61% of bitten people [4]. It has been shown that the oedematous effect induced by PNV in rats involves the generation of kinins and the stimulation of tackykinin receptors. This effect also depends on the histamine, serotonin and polypeptides contained in the venom [5], [6], [7], [8]. Because pain may occur after PNV envenomation even in the absence of oedema, the mechanisms involved in these responses could be distinct. Moreover, there are no validated therapies for treating PNV-induced pain, though local anaesthetics, opioids, non-steroidal anti-inflammatory drugs and arachnid antivenin serum have been used empirically [8], [2].

PNV contains both low- and high-molecular-weight substances that could contribute to the induction of pain [9], [3]. However, the PNV components involved in the nociceptive response as well as the molecular targets stimulated by venom components have not been identified. Thus, the aim of this study was to investigate the mechanisms involved in PNV-induced nociception in mice.

## Methods

### Ethical statements

All experiments were conducted in accordance with the current guidelines for the care of laboratory animals and the ethical guidelines for investigations of experimental pain in conscious animals [10]. All of the protocols employed were approved by the Local Ethics Committee - Comissão de Ética no Uso de Animais (process number 23081.003193/200940). The number of animals and the nociceptive stimulus used were the minimum necessary to demonstrate the consistent effects of drug treatments.

### Animals

Male Swiss mice weighing 30–35 g were maintained at 22±2°C with free access to water and food under a 12∶12 h light∶dark cycle. Animals were acclimatized in the laboratory for at least 2 h before testing and were used only once throughout the experiments. Behavioural testing was performed by an experienced observer blinded with respect to drug administration.

### Drugs

PNV was obtained by the electrical stimulation of anesthetized spiders and was kindly donated by the Fundação Ezequiel Dias (Belo Horizonte, Brazil). The arachnid antivenin (ArAv, Instituto Butatan, São Paulo, Brazil) was kindly donated by 4^a^ Coordenadoria Regional de Saúde (Santa Maria, Brazil). Aprotinin, methysergide, GR113808, ondansetron, serotonin, MK-801, DNQX, glutamate, SB366791, acetaminophen, compound 48/80, indomethacin, lidocaine, ninhydrin, tetrodotoxin, histamine, resiniferatoxin (RTX), soybean trypsin inhibitor (SBTI) and high- and low-molecular-weight human kininogen were purchased from Sigma (St. Louis, USA). Promethazine and morphine were obtained from Cristália (São Paulo, Brazil). Dipyrone was obtained from Hoechst (São Paulo, Brazil). Arachidonic acid was purchased from Cayman (Michigan, USA). Radio-labelled [^3^H]-resiniferatoxin was purchased from Perkin Elmer (Boston, USA). SR140333 and SR48968 were kindly donated by Sanofi Recherche (Paris, France).

The stock solutions of most of the drugs were prepared in phosphate-buffered saline (PBS, 137 mM NaCl and 10 mM phosphate buffer, pH 7.4, Sigma, USA) in siliconised plastic tubes, maintained at −20°C, and diluted to the desired concentration just before use. The SB366791 and resiniferatoxin stock solutions were prepared in absolute ethanol (90%) and Tween 80 (10%). The indomethacin stock solution was prepared in 5% ethanol plus 5% Tween 80. The final concentration of ethanol and Tween 80 did not exceed 0.5% in the administered drug and did not cause any detectable effects (results not shown). The stock tetrodotoxin solution was prepared in acetate buffer (100 mM, pH 4.8), and the pH of the solution was adjusted to 7.4 with Tris buffer (100 mM, pH 9.6). The pH of the glutamate solution was adjusted to 7.4 with 5 M NaOH (to a final sodium concentration of approximately 157 nM).

### Algogen-induced nociceptive and oedematogenic responses

The procedure used was similar to that described previously [11], [12], [13], [14]. The animals were placed individually in chambers (transparent glass cylinders 20 cm in diameter) and were allowed to acclimate to the tube for 20 min before treatment. A volume of 20 µl of PNV (0.3–10 µg/paw) was injected subcutaneously under the surface of the right hind paw (intraplantar, i.pl.). A separate group of animals received an i.pl. injection of the appropriate vehicle (PBS). After treatment, the mice were observed individually for 360 min.

The amount of time spent licking the injected paw was timed with a chronometer for 10 minutes and was defined as the time of nociception.

It has been reported that there are significant advantages to using a combination of several behaviours rather than a single index of pain, in terms of sensitivity and specificity [15]. Therefore, other nociceptive behaviours besides licking the affected limb were observed and categorised based on the position of the injected hind paw, and a score of 0, 1, 2 or 3 was assigned [15]. The behavioural rating criteria were as follows: (0) no pain - normal weight bearing on the injected paw; (1) favouring - resting the injected paw lightly on the floor or limping; (2) lifting - elevation of the injected paw; and (3) licking - licking or biting the injected paw. Because the mouse moves so quickly, time sampling was used to score other pain-related behaviours. Mice were observed for 5 minutes immediately after PNV or vehicle injection and also for 5 minutes at 15, 30, 60, 120, 240 and 360 min after injection.

The variation in the thickness of the paw was assessed as an oedema index [13]. The thickness was measured with a calliper immediately prior to the injection of PNV and immediately after during nociception measurement. Paw oedema was expressed as the difference (Δ) in paw thickness (mm) after PNV or vehicle injection relative to the pre-injection value for each animal.

### Sensitivity of PNV-induced nociception to clinically used analgesics

To assess the effects of clinically used analgesics on the amount of time spent licking the PNV-injected paw, venom (3 µg/paw, i.pl.) was injected into a mouse paw previously treated with morphine (10 mg/kg, i.p., 30 min prior), dipyrone (500 mg/kg, i.p., 30 min prior), acetaminophen (400 mg/kg, i.p., 30 min prior) or indomethacin (30 mg/kg, i.p., 30 min prior). PNV (3 µg/paw, i.pl.) was also co-administered with lidocaine (0.4 µmol/paw, i.pl.). The control animals received the appropriate vehicle. The dosage choice for each drug was based on previous data described in the literature [12], [16], [17] or on preliminary experiments conducted in our laboratory (data not shown).

To determine whether the nociceptive behaviour induced by PNV injection is neutralised by arachnid antivenin (ArAv), PNV was co-administered with ArAv (1∶30, dissolved in PBS). The control animals received a similar volume of boiled ArAv (1∶30, dissolved in PBS and boiled at 90°C for 15 min to inactivate the serum) or PBS (20 µl/paw). The dosage choice for ArAv was based on preliminary experiments conducted in our laboratory.

To assess the effects of clinically used analgesics on the nociceptive behaviour scores, PNV (3 µg/paw, i.pl.) was injected into the mouse paw, nociception was detected, and mice were treated with morphine (10 mg/kg, i.p., 30 min after PNV injection), dipyrone (500 mg/kg, i.p., 30 min after), acetaminophen (400 mg/kg, i.p., 30 min after), indomethacin (30 mg/kg, i.p., 30 min after), lidocaine (0.4 µmol/paw, i.pl., 55 min after) or ArAv (1∶30, i.pl., 55 min after).

### Boiling and dialysis of *Phoneutria nigriventer* venom

PNV (1 ml of 150 µg/ml diluted in PBS) was boiled for 15 min at 90°C [18]. The procedure used to dialyse PNV was similar to that described previously [5]. The PNV (2 ml of a 1 mg/ml PBS solution) was dialysed (MW cut off of the dialysis membrane was 12–14 kDa, Sigma) for 24 h at 4–6°C against 2 L of PBS. The dialysing solution was changed 7 times, at 2, 4, 6, 8, 10, 12 and 22 h.

### Estimation of histamine, serotonin and glutamate levels in the *Phoneutria nigriventer* venom

The histamine content was evaluated as previously described [19]. First, 300 µl of NaOH (1 M) was added to 1 ml of dialysed PNV or non-dialysed PNV samples and incubated with 40 µl of 1% *o*-phthaldialdehyde (OPT, Sigma, St Louis, USA) for 4 min. Then, 150 µl of HCl (3 M, pH 10.4) was added to stop the reaction and to allow the development of fluorescence. The samples were read at 360 nm (excitation) and 450 nm (emission) in a spectrofluorophotometer (RF-5301PC, Shimadzu, Japan).

The serotonin content was evaluated as previously described [20]. Dialysed PNV or non-dialysed PNV samples (0.5 ml) were incubated with 0.5 ml of ninhydrin (0.24%) and 0.5 ml of phosphate buffer (100 mM, pH 7.4). The reaction was incubated at 100°C for 10 min and allowed to rest at room temperature in the dark for 16 h. The fluorescence was read at 380 nm (excitation) and 500 nm (emission) in a spectrofluorophotometer.

The glutamate measurement was performed enzymatically following an increase in fluorescence due to the production of NADPH in the presence of glutamate dehydrogenase and NADP+ [21]. To start the assay, NADP+ (1.0 mM) and glutamate dehydrogenase (50 U) were added to the dialysed or non-dialysed PNV samples 10 min after the emitted fluorescence was measured using a spectrofluorophotometer. The excitation wavelength was set at 360 nm, and the emission wavelength was set to 450 nm.

### Measurement of kallikrein-like activity in *Phoneutria nigriventer* venom

The method used for the measurement of kallikrein activity was similar to a previously described method [22], with some modifications. The activity of a kallikrein-like enzyme was measured by the cleavage of a selective peptide-nitroanilide substrate, D-Val-Leu-Arg-paranitroanilide (D-Val-Leu-Arg-pNA) (Sigma, St. Louis, USA), which was dissolved in ultrapure water to a concentration of 1.5 mM and stored at 4°C until use. To determine the kallikrein activity, 25 µl of PNV (500, 150 or 50 µg/ml) or boiled PNV was added to 50 µl of assay buffer (0.2 M Tris-HCl, pH 8.2, containing 0.01 M EDTA) and 25 µl of substrate (D-Val-Leu-Arg-pNA, 0.375 mM) in 96-well microplates. The incubation mixtures were maintained at 37°C, and the reaction was followed by measuring the absorbance at 405 nm (measured in a Plate Reader, Bioteck, USA) from 1 to 16 min. For each experiment, we determined a standard curve using a pure synthetic form of the product generated by the reaction (p-nitroaniline, 0.003–3 mM, Merck, Darmstadt, Germany). The released p-nitroaniline was measured colorimetrically at 420 nm. The protein content of PNV was determined by the Bradford method [23] using a standard curve with known concentrations of bovine serum albumin within the absorbance range. The results of the kallikrein enzyme activity assay are expressed as nmol of the formed product (p-nitroaniline) over time (in minutes) and as the amount of protein (expressed in mg of protein) in each PNV sample [24]. To kinetically characterise the kallikrein-like activity, we incubated PNV (150 µg/ml) with different concentrations of the substrate (D-Val-Leu-Arg-pNA, 0.09–3.00 mM), and the reaction was followed by measuring the absorbance at 405 nm from 1 to 40 min. We also determined the sensitivity of the PNV enzyme to the tissue kallikrein inhibitor aprotinin and the plasma kallikrein inhibitor soybean trypsin inhibitor (SBTI). For this experiment, 25 µl of PNV (500 µg/ml) and 25 µl of the substrate (D-Val-Leu-Arg-pNA, 0.375 mM) were incubated in the presence or absence of aprotinin (10 µg/ml) or SBTI (3 µg/ml) [25]. A final volume of 100 µl was achieved by adding buffer where necessary. The reaction was followed by measuring the absorbance at 405 nm from 1 to 16 min.

### Kinin production by PNV

*Phoneutria nigriventer* venom (3 µg) was incubated with high- or low-molecular-weight human kininogen (200 nM) in 50 mM Tris buffer (pH 7.41, 0.1 M NaCl) in a final volume of 100 µL for 15 min at 37°C, as reported by [26] and [27], with minor modifications. The kinin was extracted in ice-cold ethanol (four times the final volume) and centrifuged for 60 min at 16,000× g at 4°C. The supernatants containing free kinin were collected. The solutions were freeze-dried and resuspended in 100 µl of enzyme immunoassay buffer. A kinin enzyme immunoassay was performed using a high-sensitivity kit for kinin (Bachem) according to the manufacturer's protocol. Briefly, 50 µl of the standard solutions or test samples was added to a 96-well plate. Then, 25 µl of each primary antisera and biotinylated peptide solution were added, and the plates were incubated for 2 h at room temperature with mild agitation. The plates were then washed five times, and 100 µl of a diluted streptavidin-conjugated horseradish peroxidase solution was added to each well. After a 60-min incubation at room temperature, the immunoplates were washed five times, and 100 µl of a 3,3′,5,5′-tetramethyl benzidine dihydrochloride (TMB) solution was added to each well. After an additional 20-min incubation at room temperature, the reaction was stopped with 100 µl of 2 N HCl. The absorbance was read at 60 min after stopping the reaction at 490 nm. The blank control consisted of 100 µl of TMB solution and 100 µl of 2 N HCl.

### Pharmacological study of the peripheral mechanisms involved in PNV-induced nociception

To assess the involvement of peripheral mechanisms in the nociceptive responses induced by PNV (3 µg/paw), animals were co-injected with the non-selective 5-HT receptor antagonist methysergide (10 nmol/paw), the selective 5-HT_4_ receptor antagonist GR113808 (15 nmol/paw), the selective 5-HT_3_ receptor antagonist ondansetron (30 nmol/paw), the H_1_ receptor antagonist promethazine (3 nmol/paw), the AMPA/kainate receptor antagonist DNQX (1 nmol/paw), the selective NMDA receptor antagonist MK-801 (1 nmol/paw), the tissue kallikrein inhibitor aprotinin (100 µg/paw), the plasma kallikrein inhibitor soybean trypsin inhibitor (SBTI, 3 µg/paw), the B_2_ receptor antagonist HOE 140 (3 nmol/paw), the ASIC blocker amiloride (100 nmol/paw), the selective NK_1_ antagonist SR14333 (0.2 nmol/paw), the selective NK_2_ antagonist SR48968 (20 nmol/paw), the selective TRPV1 receptor antagonist SB366791 (1 nmol/paw), the cyclooxygenase inhibitor indomethacin (30 µmol/paw) or the Na^+^ channel blocker tetrodotoxin (20 pmol/paw). The control animals received a similar volume of the appropriate vehicle or PBS (20 µl/paw). To determine the efficacy or the nonspecific effects of the tested doses for each antagonist, we tested the effect of GR113809 (30 nmol/paw), ondansetron (30 nmol/paw) or methysergide (10 nmol/paw) co-administered with serotonin (100 nmol/paw), MK-801 (1 nmol/paw) or DNQX (1 nmol/paw) co-administered with glutamate (10 µmol/paw), indomethacin (30 µmol/paw) co-administered with arachidonic acid (100 nmol/paw) or prostaglandin E_2_ co-administered with tetrodotoxin (20 pmol/paw). The dose of each drug was based on previous data described in the literature [28], [29], [30], [31], [32] or on preliminary experiments conducted in our laboratory (data not shown).

To explore the participation of mast cells in PNV-induced nociception, the mast cell degranulation compound 48/80 was administered daily at increasing doses (1, 3, 10 and 10 µg/paw, i.pl.) as described previously [12].

To further explore the role of TRPV1-positive sensory fibres in the nociception induced by PNV, animals were subjected to a systemic desensitisation protocol as previously described [33]. The animals were anesthetised with isoflurane and received systemic administration of resiniferatoxin (50 µg/kg) or the vehicle alone (0.5% ethanol, 0.5% Tween-80, PBS). After 7 days, the animals received a subcutaneous injection of PNV (3 µg/paw, i.pl.), capsaicin (1 nmol/paw, used as a positive control) or vehicle (0.5% ethanol, 0.5% Tween-80 and PBS; 20 µl/paw), and the nociceptive behaviour was evaluated.

### [^3^H]Resiniferatoxin binding assay

Binding assays were conducted as described previously [34]. To obtain membranes for the binding studies, the spinal cords of mice were removed and disrupted with a tissue homogeniser in ice-cold buffer A (pH 7.4), which contained 5 mM KCl, 5.8 mM NaCl, 2 mM MgCl_2_, 0.75 mM CaCl_2_, 12 mM glucose, 137 mM sucrose and 10 mM HEPES. The homogenate was first centrifuged for 10 min, 1,000× g at 4°C. The low-speed pellets were discarded, and the supernatants were further centrifuged for 30 min, 35,000× g at 4°C. The resulting high-speed pellets were re-suspended in buffer A and stored at −20°C until assayed. The binding assays were conducted in duplicate with a final volume of 500 µl, which contained buffer A supplemented with 0.25 mg/ml bovine serum albumin, membranes (100 g/protein) and 50 pM [^3^H]-resiniferatoxin in the presence or absence of PNV (1.5–150 µg/ml). To measure nonspecific binding, 100 nM of non-radioactive RTX was included. The assay mixtures were set up on ice, and the binding reaction was initiated by transferring the assay tubes to a 37°C water bath. After a 60-min incubation period, the mixtures were cooled on ice to terminate the binding reaction. Then, 100 mg of bovine α1-acid glycoprotein (Sigma, St. Louis, USA) was added to each tube to reduce nonspecific binding. Finally, the bound and free membranes of [^3^H]-resiniferatoxin were separated by centrifugation for 15 min, 20,000× g at 4°C. The pellet was quantified using scintillation counting. The specific binding was calculated as the difference between the total and nonspecific binding.

### HEK293 cell culture and transfection

HEK293 cells were grown in 89% DMEM, 10% FBS, and 1% of an aqueous solution containing penicillin (5000 units/mL) and streptomycin (5000 µg/mL) (Gibco). The cells were resuspended and passaged every 5–6 days. The plasmid containing rat-TRPV1 was kindly provided by Dr. David Julius (UCSF/CA/USA). After growing to 80–90% confluence, the cells were transfected with rTRPV1using Lipofectamine 2000 (Invitrogen) according to the manufacturer's protocol. Calcium imaging experiments were performed 12–24 h after the transfection.

### Calcium fluorescence imaging

The experiments were performed at room temperature (20–25°C), essentially as described previously [35]. The cells on cover slips were treated with 3 µM Fluo 4-AM (Molecular Probes) for 40 min in HEPES-buffered salt solution (HBSS) containing (in mM) 124 NaCl, 4 KCl, 1 CaCl2, 1.2 MgCl2, 10 glucose, and 25 HEPES, at pH 7.4. The cover slips were washed in dye-free HBSS, transferred to a superfusion chamber system (Bioptechs) and placed on the stage of a microscope. The HBSS solution (alone or with the treatments) was continuously perfused (0.6 ml/min) via a peristaltic pump (Bioptechs) during image acquisition. Calcium imaging was performed with a Leica SP5 laser scanning confocal system using LAS software with a 10× objective lens. Fluo 4-AM was imaged by excitation with a 488-nm argon laser line, and the emitted light was collected at a 510–570 nm emission band. The cells were stimulated for 20 s with HBSS containing capsaicin or PNV at different concentrations. Quantitative measurements were made by re-analysing the stored image sequences using LAS Application software. Regions of interest (ROI) were identified within the soma of individual cells of at least 3 independent plates. Changes in fluorescence (F) were normalised to the initial fluorescence (F0) for each cell and expressed as (F/F0)×100 (% of baseline). For dose-response curves, the average amplitude of the [Ca^2+^] changes represents the difference between the baseline and the transient peak of fluorescence in response to capsaicin or PNV. A control group, prepared by Lipofectamine transfection without plasmid, is indicated in the text as non-transfected cells.

### Statistical analysis

The results are presented as the mean±S.E.M. except for the ED_50_ values (i.e., the dose of agonist necessary to produce 50% of the pain response relative to the maximum effect), which are reported as geometric means accompanied by their respective 95% confidence limits, and the spontaneous nociception scores, which were reported as medians and interquartile ranges. The ED_50_ values were calculated using linear regression for individual experiments in the GraphPad Prism software. Spontaneous nociception scores were analysed using the Mann-Whitney U test. All other data were analysed using the Student's t-test, one-way ANOVA followed by Dunnett's test or two-way ANOVA followed by Bonferroni's test, as appropriate. P values of less than 0.05 were considered to be significant.

## Results

### *Phoneutria nigriventer* venom induced ongoing nociception and oedema

**Figure 1:**
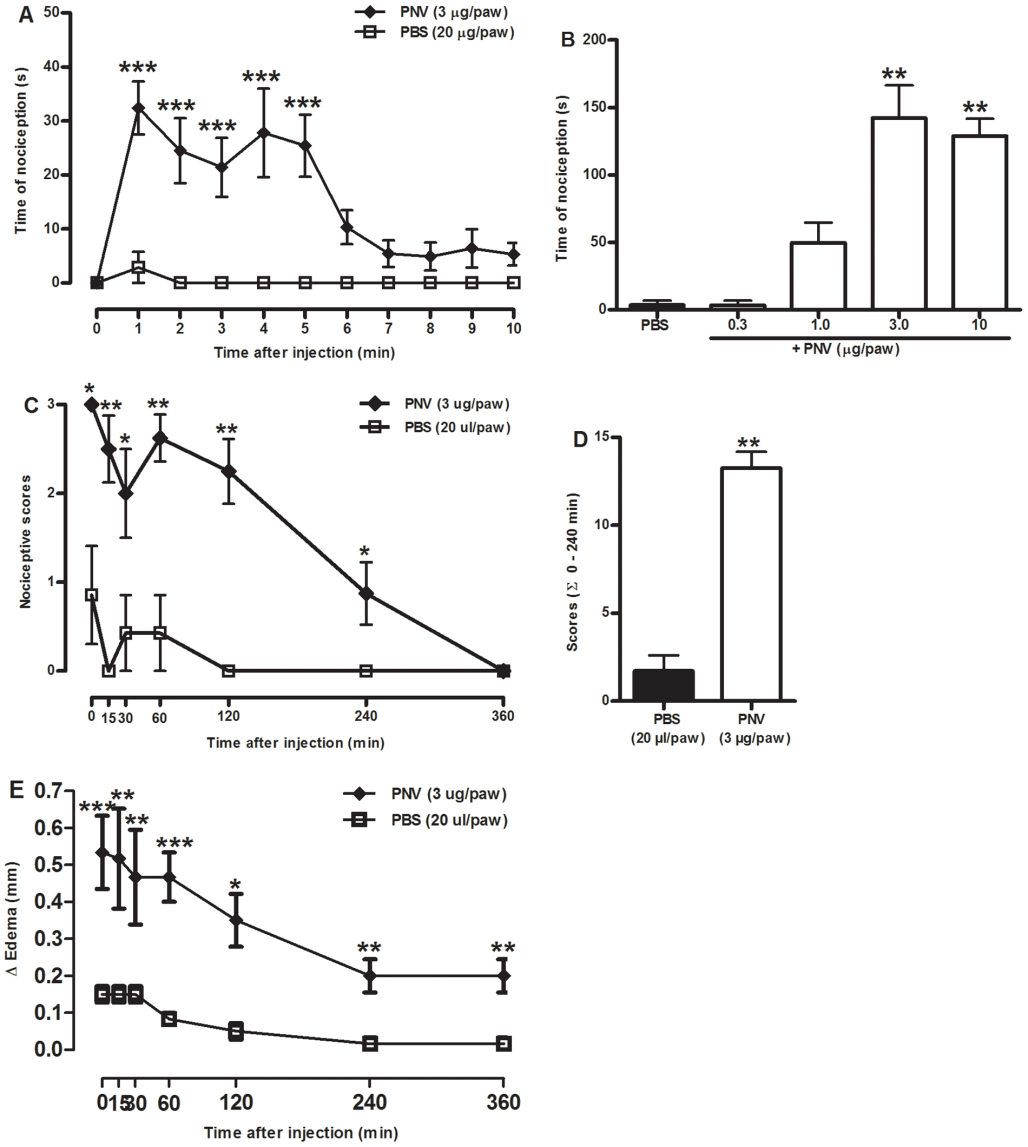
Nociception and oedema induced by i.pl. injection of PNV in mice. (A–B) Time-course (A) and dose-response (B) curves for the ongoing nociception, measured as an increase in amount of time spent licking the injected paw, induced by PNV. (C–D) Time-course (C) and sum (Σ, D) of the pain-related behaviour scores from 0 to 240 minutes after PNV injection. (E) Time-course of the paw oedema caused by i.pl. injection of PNV in mice. Each point or bar on the curve represents the mean±S.E.M or the medians and interquartile ranges of 5–7 animals. The asterisks denote the significance levels. **P<0.01, ***P<0.001 compared with the PBS group (A, E: two-way ANOVA followed Bonferroni's test, B: one-way ANOVA followed by Dunnett's test, C, D: Mann-Whitney U test).

The subcutaneous administration of PNV (0.3–10 µg/paw) in mice produced dose-dependent, continuous nociception, verified as an increase in the amount of time spent licking the injected paw (Figure 1A–B). This behaviour occurred quickly, peaked from 1 to 5 min and declined 6 min after the PNV injection (Figure 1A). The calculated ED_50_ value of PNV was 1.6 (0.8–2.4) µg/paw, and the maximum licking response time was 147.5±17.0 s.

In addition to licking, other nociceptive behaviours (favouring and lifting) were observed and scored according to the position of the injected hind paw. When a combination of behaviours was assessed, PNV (3 µg/paw) was capable of producing pain-related behaviour up to 4 hours after the injection (Figure 1C). The means of sum of the behaviour scores from 0 to 240 min after PNV injection were 2 and 13 for vehicle and PNV, respectively (Figure 1D). PNV injection also produced a marked paw oedema that was detected as early as 5 min after the injection and was reduced, but still significant, 4–6 hours after injection (Figure 1E).

### Clinically used analgesics reduced PNV nociception

**Figure 2:**
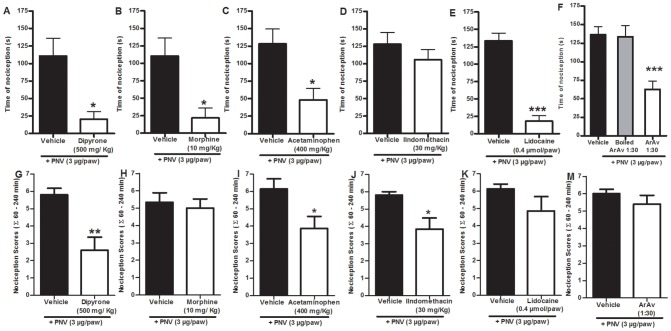
Clinically used analgesics reduced PNV nociception. The effect of systemic pre- (A–D) or post-treatment (G–J) with dipyrone (500 mg/kg, i.p., A and G), morphine (10 mg/kg, i.p., B and H), acetaminophen (400 mg/kg, i.p., C and I) or indomethacin (30 mg/kg, i.p., D and J) on PNV-induced (3 µg/paw) nociception in mice. The effect of local co-administration (E–F) or post-treatment (K–M) with lidocaine (0.4 µmol/paw, i.pl., E and K) or arachnid antivenin (ArAv 1∶30, i.pl., F and M) on PNV-induced (3 µg/paw) nociception. Each column represents the mean or median of 6–8 animals, and the vertical lines show the S.E.M. or interquartile ranges *P<0.05, **P<0.01 ***P<0.001 compared with the vehicle group. A–E: Student's t-test and G–M: Mann-Whitney U test, F: one-way ANOVA followed by Dunnett's test.

The systemic pre-administration of morphine (10 mg/kg, i.p., Figure 2B), dipyrone (500 mg/kg, i.p., Figure 2A) or acetaminophen (400 mg/kg, i.p., Figure 2C), but not indomethacin (30 mg/kg, i.p., Figure 2D), partially reduced the time of nociception caused by PNV (3 µg/paw) injection. The calculated percentages of inhibition were 80±12, 81±10 and 62±13%, respectively. Moreover, the local co-administration of lidocaine (0.4 µmol/paw) or ArAv (1∶30) was able to reduce the PNV-induced nociception with an inhibition of 86±6 and 54±8%, respectively, compared to the control group (Figure 2E and F).

To mimic a clinical setting, PNV (3 µg/paw) was injected, nociception scores were observed up to 30 min, and a clinically used analgesic was administered followed by observation of nociception again for 60 to 240 minutes after PNV injection. Similar to the results of pre-treatment, post-treatment with dipyrone and acetaminophen were capable of reducing nociception scores, though less efficaciously than pre-treatment (55±13% and 39±9% inhibition, respectively) (Figure 2G, I). In contrast to the pre-treatment results, post-treatment with neither morphine, lidocaine nor ArAv was unable to alter the nociception scores (Figure 2H, K and M), though indomethacin inhibited nociception (inhibition of 34±10%) (Figure 2J).

To avoid unnecessary long-lasting animal discomfort and focus on the mechanisms responsible for the development, but not the maintenance, of nociception trigged by PNV, the dose of 3 µg/paw and the time spent licking the injected paw were chosen to study a subset of mechanisms involved in PNV-induced nociception.

### Effect of boiled or dialysed venom in PNV-induced nociception

**Figure 3:**
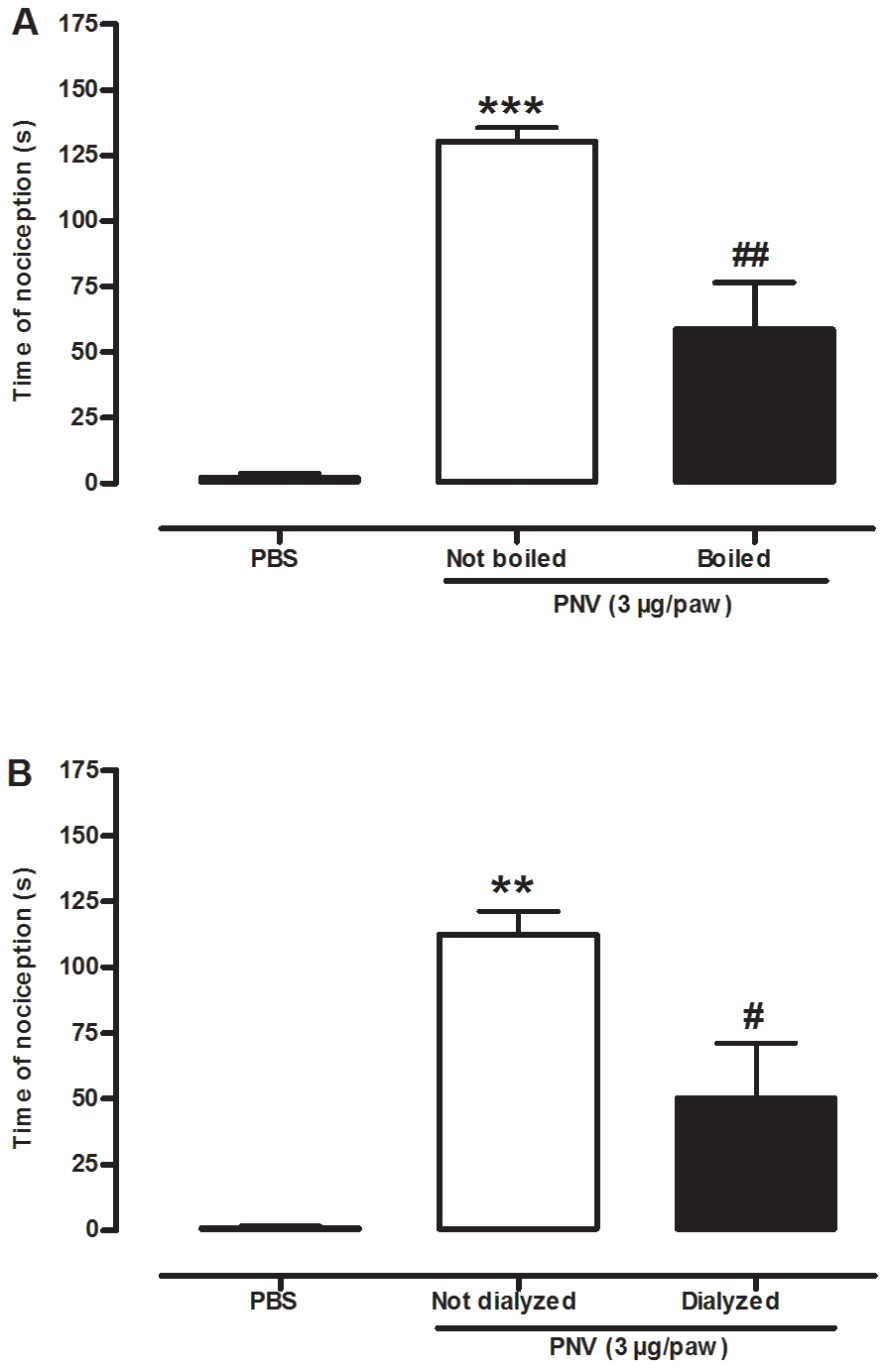
Effect of boiling or dialysing venom in the PNV-induced nociception. The nociceptive effect produced by i.pl. injection of boiled (A) or dialysed (B) PNV (3 µg/paw) in mice. Each column represents the mean± S.E.M of 6 mice. The asterisks denote the significance levels. **P<0.01, ***P<0.001 compared with the PBS group. ^#^P<0.05, ^##^P<0.01 compared with the non-boiled or non-dialysed PNV group (one-way ANOVA followed by the Student-Newman-Keuls test).

To determine whether the thermolabile and low-molecular-weight substances in PNV are related to venom-induced nociception, animals received a s.c. injection of boiled or dialysed PNV (3 µg/paw). The spontaneous nociception trigged by native PNV was significantly reduced by boiling (55±14%) or dialysis (55±18%) (Figure 3A and 3B). These results suggest that PNV-induced nociception was induced by the combined action of a thermolabile substance (possibly with a high molecular weight) as well as low-molecular-weight (and thermal-resistant) substances.

### Role of glutamate, histamine, serotonin and mast cells in the nociceptive effect triggered by PNV

We were able to identify the pro-inflammatory substances histamine, serotonin and glutamate in PNV (Table 1). The concentrations of these substances were largely reduced by venom dialysis (Table 1). We next verified the effect of serotonin, histamine and glutamate receptor antagonists on PNV-induced nociception.

**Table 1:** Amount of histamine, serotonin and glutamate in PNV.

Substances	Non-dialysed PNV (µg/ml)	Dialysed PNV (µg/ml)
Histamine	0.036	0.008
Serotonin	0.23	0.08
Glutamate	1.23	0.44

**Figure 4:**
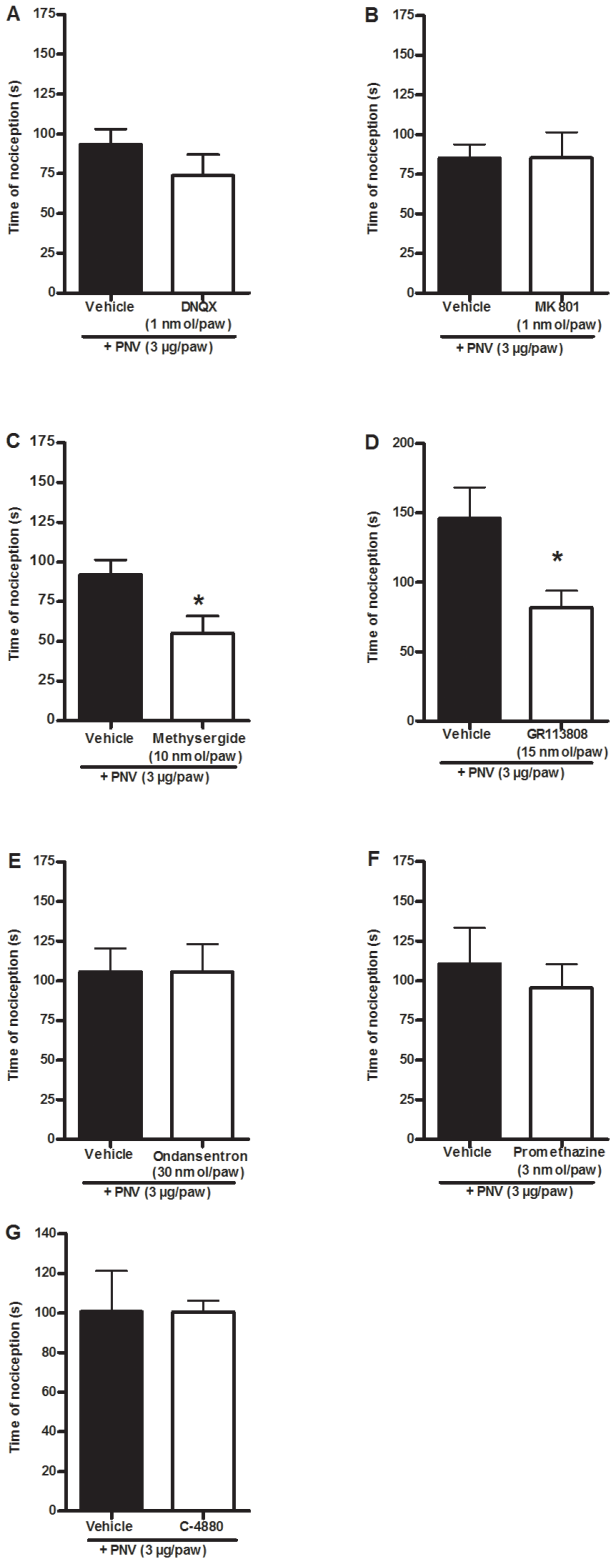
Role of glutamate, histamine, serotonin and mast cells in the nociceptive effect triggered by PNV. The effect of i.pl. treatment with the AMPA/kainate receptor antagonist DNQX (1 nmol/paw, A), the NMDA receptor antagonist MK-801 (1 nmol/paw, B), the 5-HT receptor antagonist metysergide (10 nmol/paw, C), the 5-HT_4_ receptor antagonist GR113808 (15 nmol/paw, D), the 5-HT_3_ receptor antagonist ondansetron (30 nmol/paw, E), the H_1_ receptor antagonist promethazine (3 nmol/paw, i.pl., F) or pre-treatment with compound 48/80 (1, 3, 10 and 10 µg/paw, i.pl., G) on PNV-induced (3 µg/paw, i.pl.) nociception in mice. Each column represents the mean± S.E.M of 6–8 mice. The asterisks denote the significance levels. *P<0.05 compared with the vehicle group (Student's t-test).

Regarding the role of glutamate, we observed that the co-administration of either the AMPA/kainate receptor antagonist DNQX or the selective NMDA receptor antagonist MK-801 (both at 1 nmol/paw, Figure 4A and 4B) was not able to alter the nociceptive effect trigged by PNV (3 µg/paw). However, the co-administration of DNQX (1 nmol/paw) or MK-801 (1 nmol/paw) reduced the nociception caused by glutamate (10 µmol/paw), with inhibition values of 55±6 and 47±12%, respectively (Table S1).

The s.c. co-administration of the non-selective 5-HT receptor antagonist methysergide (10 nmol/paw, Figure 4C) or the selective 5-HT_4_ receptor antagonist GR113808 (15 nmol/paw, Figure 4D) decreased PNV-induced nociception (3 µg/paw) with inhibitions of 40±12% and 44±8%, respectively, compared to the control group (Figure 4). The selective 5-HT_3_ receptor antagonist ondansetron (30 nmol/paw, Figure 4E) did not inhibit nociception. The co-administration of methysergide (10 nmol/paw) or ondansetron (30 nmol/paw) reduced the nociception caused by serotonin (100 nmol/paw), with inhibitions of 98±2% and 89±6%, respectively (Table S1). Interestingly, the co-administration of GR113808 (15 nmol/paw) was not able to reduce the nociceptive effect induced by serotonin (100 nmol/paw) (Table S1).

The histamine H_1_ receptor antagonist promethazine (3 nmol/paw, Figure 4F) did not alter PNV-induced nociception (3 µg/paw). In addition, we observed that the i.pl. injection of a high dose of histamine (100 nmol/paw) did not produce nociception in mice, but did induce a paw oedema that was reduced by co-administration with promethazine (3 nmol/paw, data not shown).

Moreover, the degranulation of mast cells, which can release histamine and serotonin, was not necessary to induce nociception, given that a previous degranulation of the mast cells did not reduce PNV-induced nociception (3 µg/paw, Figure 4G) but was able to prevent compound 48/80-induced nociception (Table S1).

### Role of tissue kallikrein-like activity in the nociceptive effect trigged by PNV

**Figure 5:**
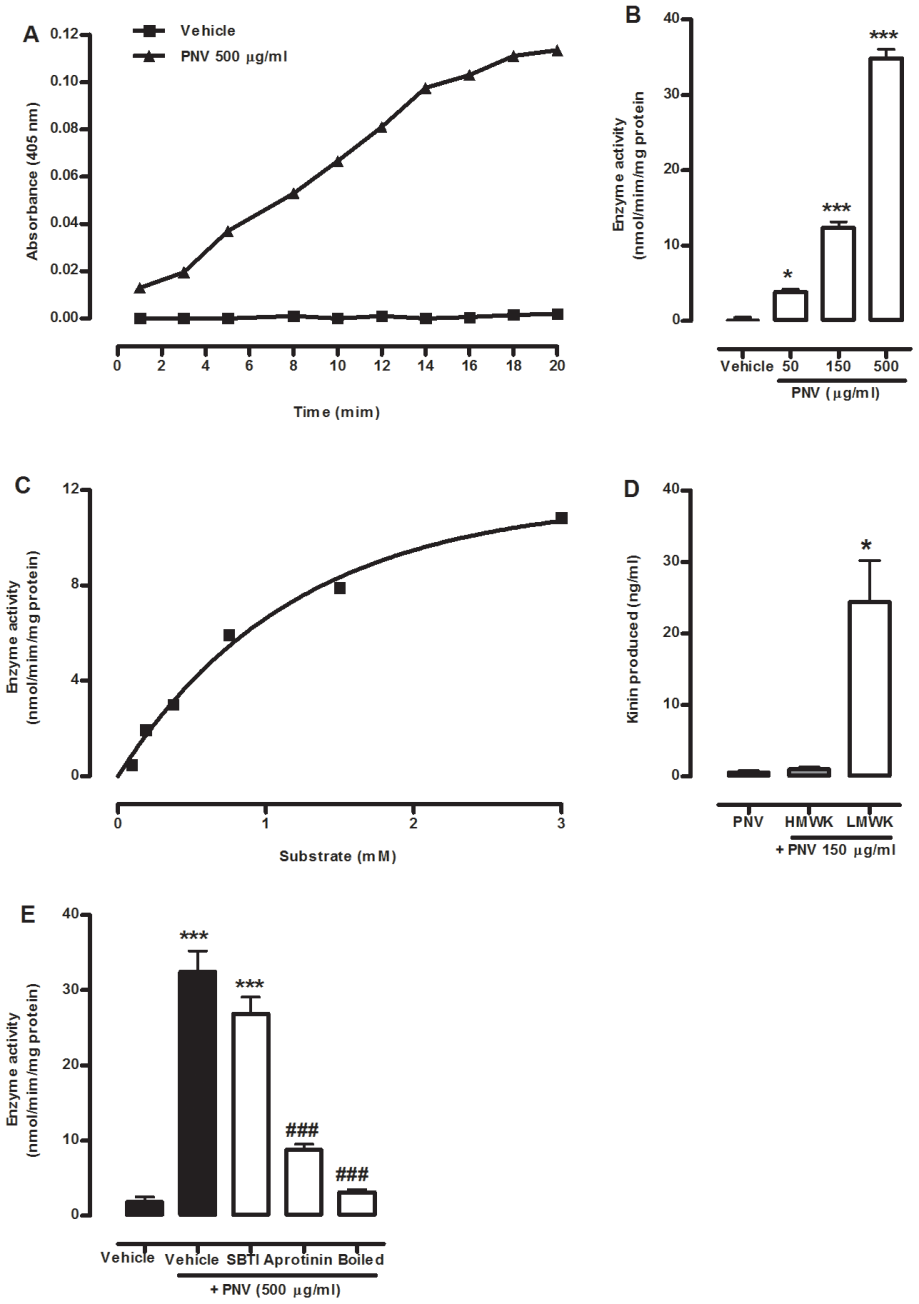
The tissue kallikrein-like activity of PNV *in vitro*. The time-course (A) and concentration-response (B) curves of tissue kallikrein-like activity in PNV. (C) The substrate (D-Val-Leu-Arg p-nitroaniline, 0.09–3.00 mM) concentration curve for the kallikrein-like activity of PNV (150 µg/ml). (D) Kinin detection after the incubation of PNV (150 mg/ml) with human high- (HMWK) or low- (LMWK) molecular-weight kininogen (200 nM). (E) The kallikrein-like activity of PNV in the presence or absence of the plasma kallikrein inhibitor SBTI (3 µg/ml) or the tissue kallikrein inhibitor aprotinin (10 µg/ml) or after boiling. Each point or bar represents the mean±S.E.M of 3 experiments carried out in duplicate. The asterisks denote the significance levels. *P<0.05, ***P<0.001, compared with the vehicle group. ^###^P<0.001 compared with the vehicle plus PNV group (one-way ANOVA followed by the Student-Newman-Keuls test).

We detected the tissue kallikrein-like activity of PNV *in vitro* by measuring the hydrolysis of the selective tissue kallikrein substrate D-Val-Leu-Arg p-nitroaniline. We found that the incubation of PNV with the substrate produced a tissue kallikrein-like activity that increased with time and was linear from 1 to 16 min after incubation (Figure 5A). Different concentrations of PNV (50–500 µg/ml) produced a kallikrein-like activity that was concentration dependent (Figure 5B). Furthermore, the substrate kinetic curve (0.09–3 mM) demonstrated that the tissue kallikrein-like enzyme in PNV (150 µg/ml) had a Michaelis-Menten constant (K_M_) value of 1.47±0.09 mM and a maximal velocity (V_max_) of 16.13±0.62 nmol/min/mg protein (Figure 5C). Moreover, the kallikrein-activity of PNV (500 µg/ml) was inhibited by boiling PNV or by the tissue kallikrein inhibitor aprotinin (10 µg/ml) but not by the plasma kallikrein inhibitor SBTI (3 µg/ml) (Figure 5E).

Because PNV demonstrated kallikrein-like activity, we next determined the ability of the venom to generate kinins from natural kininogen substrates. The results presented in Figure 5D show that the incubation of PNV (150 µg/ml) with low- but not high-molecular-weight kininogens resulted in kinin production.

**Figure 6:**
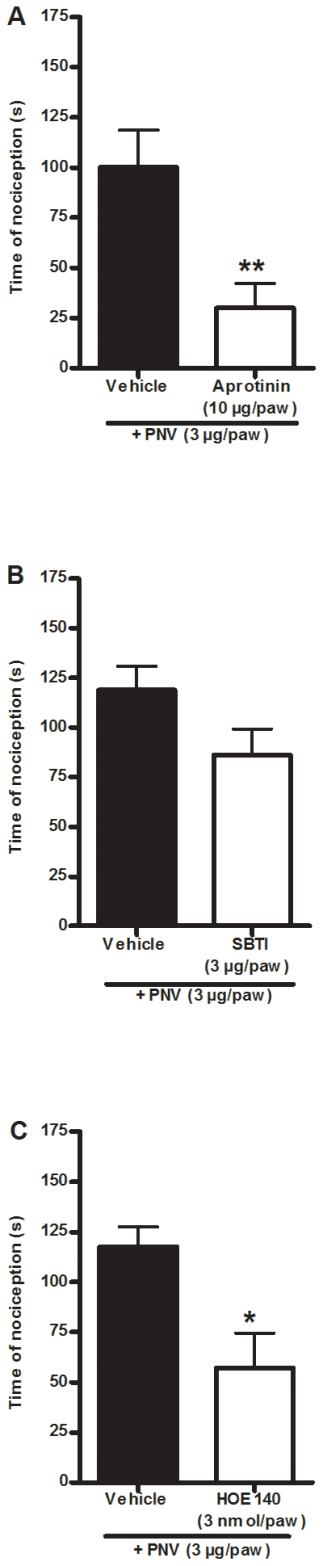
Role of tissue kallikrein and kinins in PNV-induced nociception. The effect of i.pl. treatment with the kallikrein inhibitors aprotinin (10 µg/paw, A) and SBTI (3 µg/paw, B) or the B_2_ receptor antagonist HOE140 (3 nmol/paw, D) on PNV-induced (3 µg/paw, i.pl.) nociception in mice. Each column represents the mean± S.E.M of 5–6 mice (A, B and C). The asterisks denote the significance levels. *P<0.05, **P<0.01 compared with the PNV group (A, B, C: Student's t-test).

We next investigated whether the tissue kallikrein-like activity observed *in vitro* was related to the nociception produced by the venom *in vivo*. I.pl. co-administration with aprotinin (100 µg/paw, Figure 6A), but not the soybean trypsin inhibitor (3 µg/paw, Figure 6B), reduced the PNV-induced nociception with an inhibition of 70±12% compared to the control group. These results indicate that the *Phoneutria nigriventer* venom has a tissue kallikrein-like activity that is important to its nociceptive action.

Tissue kallikrein generates kinins that may stimulate the B_2_ kinin receptor to induce pain [36]. Indeed, the i.pl. co-administration of the selective B_2_ receptor antagonist HOE 140 (3 nmol/paw, Figure 6D) with PNV (3 µg/paw) reduced the nociceptive effect induced by PNV with an inhibition of 43±14%. These data demonstrate that the tissue kinins generated by PNV play a critical role in the nociceptive effect of PNV.

### Additional investigation of the mechanisms involved in PNV-induced nociception

The serotonin or kinin action on the metabotropic 5-HT_4_ or B_2_ receptors that induces sensory fibre depolarisation and consequently triggers nociception depends on the stimulation of several enzymes and ion channels. Thus, we next investigated some of these targets in the nociceptive behaviour produced by PNV.

**Figure 7:**
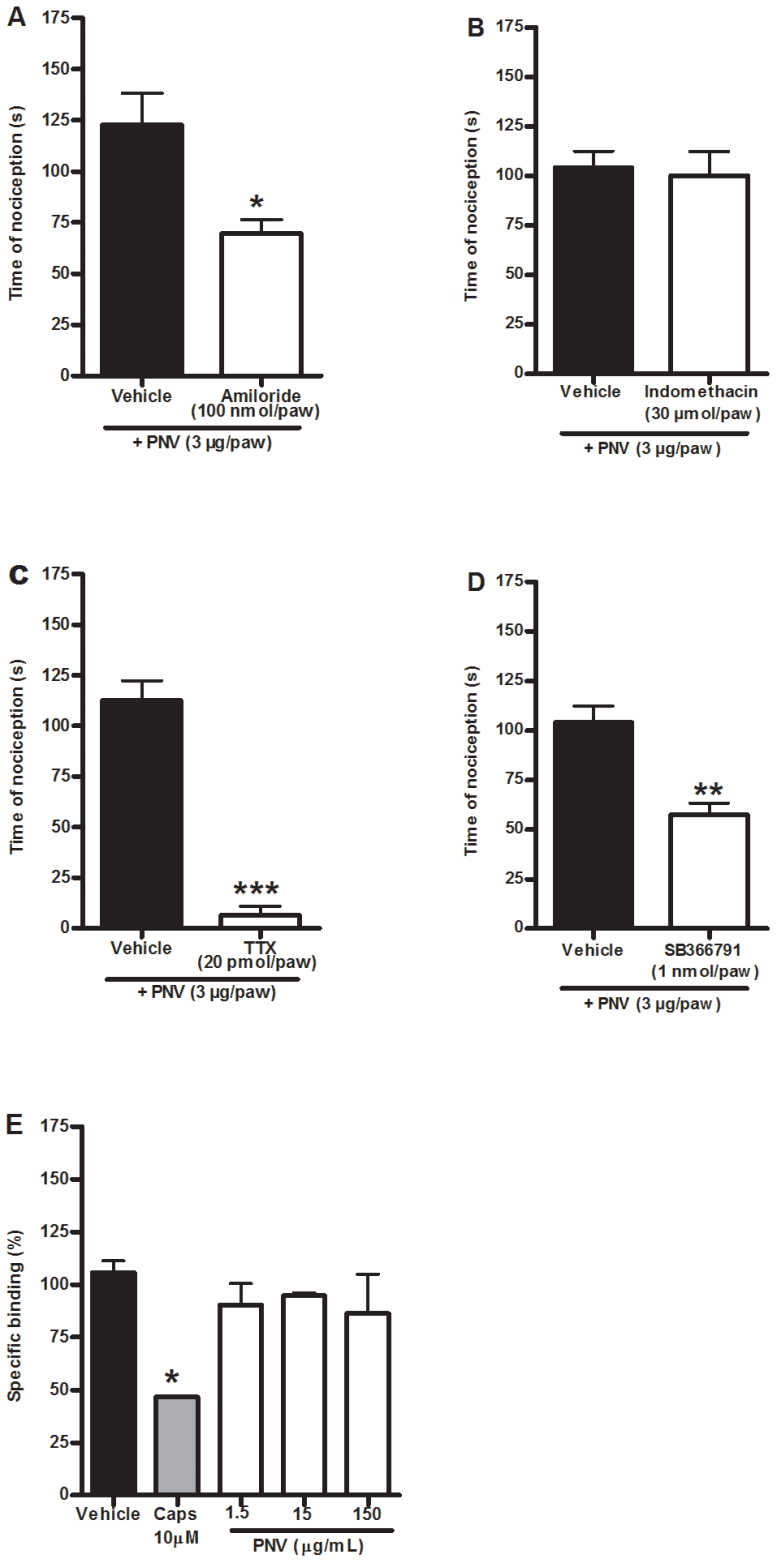
Involvement of TRPV1, ASIC, cyclooxygenase or sodium channels in PNV-induced nociception. The effect of i.pl. treatment with the acid-sensitive ion channel (ASIC) blocker amiloride (100 nmol/paw, A), the cyclooxygenase inhibitor indomethacin (30 µmol/paw, B), the sodium channel blocker tetrodotoxin (TTX) (20 pmol/paw, C) or the selective TRPV1 antagonist SB366791 (1 nmol/paw, D) on PNV-induced (3 µg/paw, i.pl.) nociception in mice. E) The specific binding of [^3^H]-resiniferatoxin to spinal cord membranes in the presence or absence of capsaicin (10 µM) or PNV (1.5–150 µg/ml). Each column represents the mean± S.E.M of 5–6 mice (A–D) of 3 experiments carried out in duplicate (E). The asterisks denote the significance levels. *P<0.05, ***P<0.001, compared with the vehicle group (A–D: Student's t-test, E: one-way ANOVA followed by the Student-Newman-Keuls test).

To test the possible involvement of the TRPV1 receptor, ASIC receptor, cyclooxygenase and sodium channels, mice received i.pl. co-administration of the ASIC blocker amiloride (100 nmol/paw, Figure 7A), the cyclooxygenase inhibitor indomethacin (30 µmol/paw, Figure 7B), the sodium channel blocker tetrodotoxin (20 pmol/paw, Figure 7C) or the selective TRPV1 antagonist SB366791 (1 nmol/paw) with PNV (3 µg/paw, Figure 7D). The results demonstrate that amiloride, tetrodotoxin and SB366791, but not indomethacin, reduced PNV-induced nociception with inhibitions of 43±5%, 94±4% and 45±6%, respectively. To confirm its general anti-nociceptive activity at the tested dose, indomethacin (30 µmol/paw) was co-administered with arachidonic acid (100 nmol/paw, Table S1), which reduced nociception by 71±19%. Tetrodotoxin was not capable of reducing PGE2-induced nociception, demonstrating that its effect is not unspecific (Table S1). These results show that the ASIC receptor, the tetrodotoxin-sensitive sodium channels and the TRPV1 receptor are some of the mechanisms underlying the nociceptive action of PNV.

It has been recently demonstrated that spider venoms may contain toxins that directly bind to and stimulate the TRPV1 receptor [37], [38], [39]. It appears that PNV does not possess such toxins because high concentrations of PNV (1.5–150 µg/ml, Figure 7E) were not able to alter the specific binding of the TRPV1 ligand [^3^H]-resiniferatoxin *in vitro*. Under the same conditions, capsaicin (10 µM, Figure 7E) inhibited the specific binding of [^3^H]-resiniferatoxin to membranes (inhibition of 56.0±0.3% compared to the control group).

**Figure 8:**
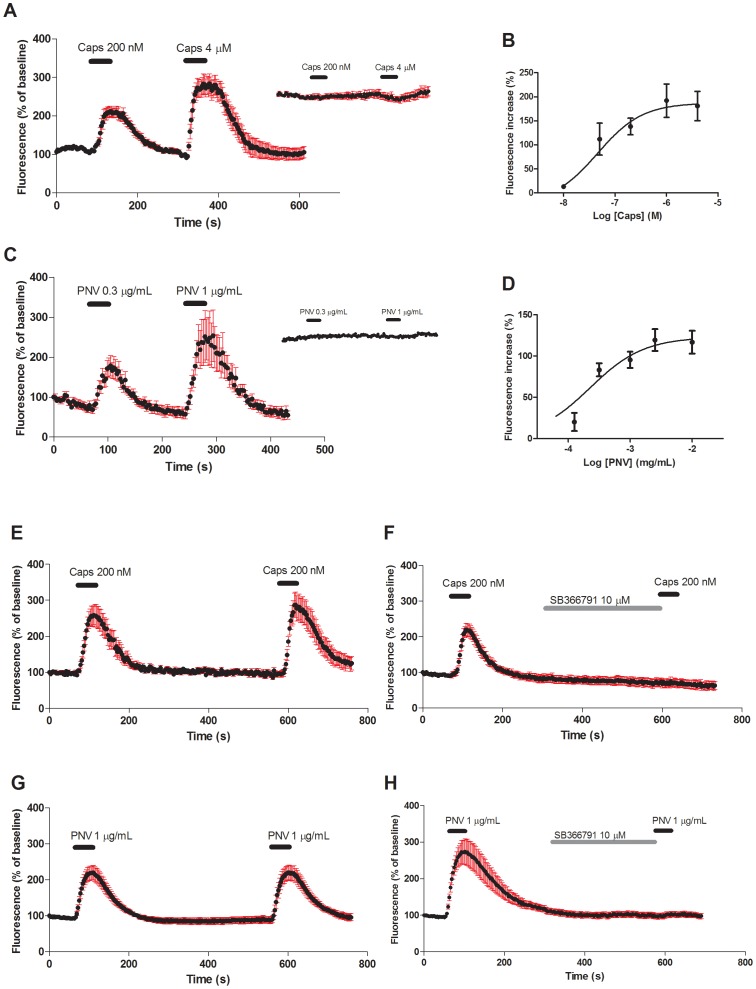
PNV effect on functional TRPV1 channels. Normalised levels of fluorescence over time from HEK293 cells transfected with rat TRPV1 cDNA and stained with the fluorescent Ca^2+^ probe fluo-4/AM. Capsaicin (A) or PNV (C) were applied in 20 s pulses as indicated by the horizontal bars above the graphs. No Ca^2+^ transient signals were observed for capsaicin or PNV addition in non-transfected cells (insets in A and C). Dose-response curves for capsaicin (B) or PNV (D) on transient calcium signals. When applied to cells twice, both capsaicin (200 nM) (E) and PNV (1 µg/mL) (G) elicited Ca^2+^ transient signals with similar amplitudes. Such response was totally abolished by pre-incubation (300 s) with 10 µM SB366791 (F and H). Points denote mean±S.E.M. of representative plates (10–30 cells, 3 independent experiments).

We further investigated a possible role of PNV on functional TRPV1 channels. Calcium levels in HEK293 cells transiently expressing TRPV1 were monitored by using the fluorescent calcium probe Fluo 4-AM. Functional expression of TRPV1 was detected by brief Ca^2+^ transient signals observed following short pulses (20 s) of capsaicin at different doses (Figure 8A–B) with an EC_50_ of 46 (7–288) nM. Similarly, PNV (20 s pulses) also induced dose-dependent Ca^2+^ transient signalling in HEK293-TRPV1 cells (Fig. 8C–D), with an EC_50_ value of 0.22 (0.03–1.81 µg/mL). No transient Ca^2+^ signal was observed in non-transfected cells after capsaicin or PNV application (Fig. 8A and C, inserts). Following the first transient Ca^2+^ signal, both capsaicin (Fig. 8E) and PNV (Fig. 8G) could elicit a new signal in HEK293-TRPV1 with similar amplitudes to the respective initial stimulus. However, incubation (300 s) with the selective TRPV1 antagonist SB366791 (10 µM) totally inhibited the second response elicited by capsaicin (Fig. 8F) or PNV (Fig. 8H). Taken together, these data show that PNV functionally interacts with TRPV1 to elicit transient calcium signalling in cells expressing these channels.

**Figure 9:**
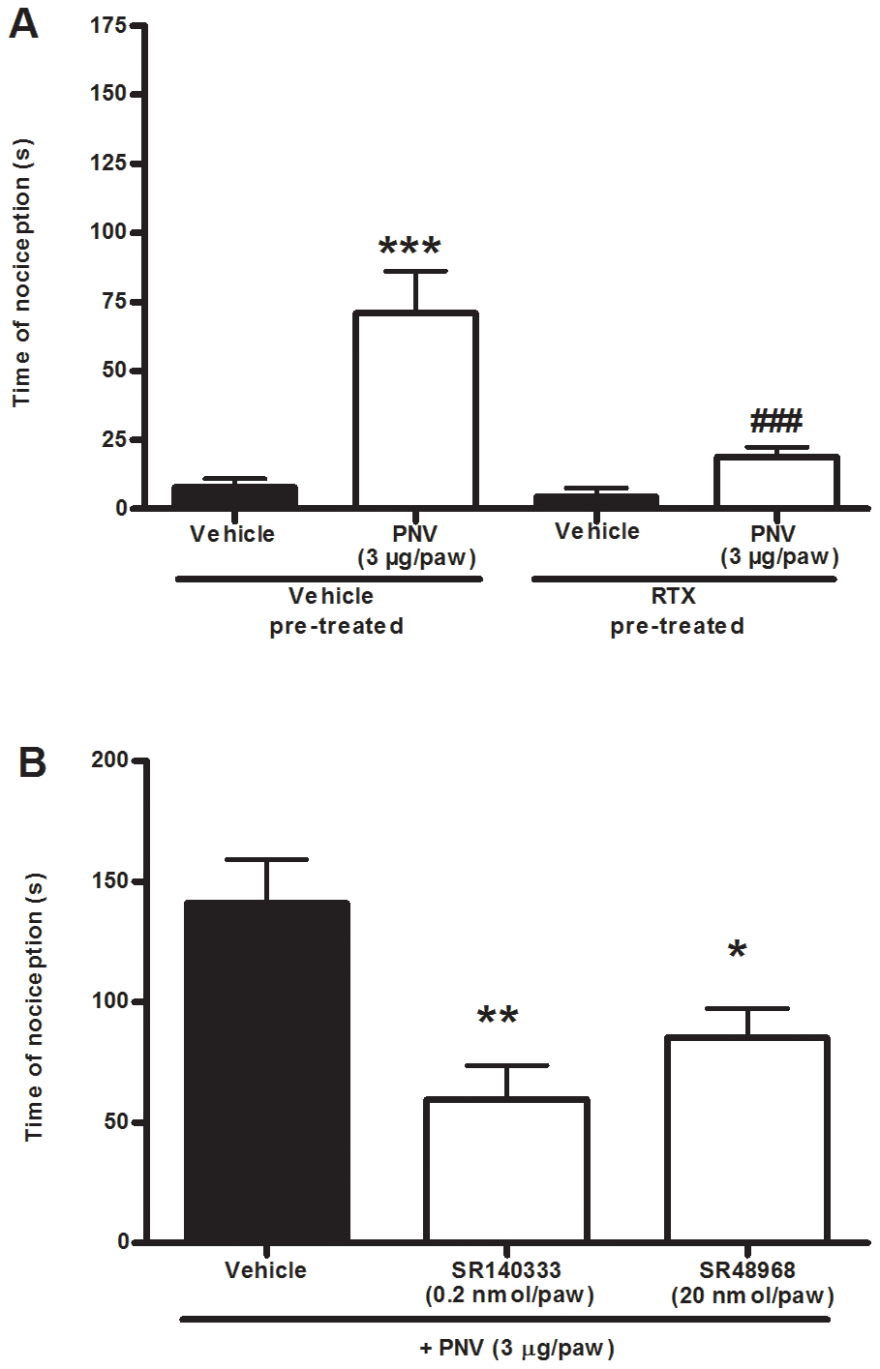
The role of TRPV1-expressing sensory fibres or tackykinin NK_1_ or NK_2_ receptors in PNV-induced nociception. The effect of TRPV1-positive sensory fibre ablation with a systemic injection of resinferatoxin (50 mg/kg, A) or the co-administration of the selective antagonists of tackykinin (B) NK_1_ (SR143033, 0.2 nmol/paw) or NK_2_ (SR48968, 20 nmol/paw) receptors on PNV-induced (3 µg/paw, i.pl.) nociception in mice. Each column represents the mean±S.E.M of 5–7 mice. The asterisks denote the significance levels. ***P<0.001 in comparison to the vehicle plus vehicle group (A), ^###^P<0.001 difference in comparison to the PNV plus vehicle treated group (A), *P<0.05, **P<0.01, in comparison to the vehicle plus PNV treated group (B) (one-way ANOVA followed by the Student-Newman-Keuls test).

To investigate the role of TRPV1-expressing sensory fibres in the PNV-induced nociception, animals were subjected to a resiniferatoxin ablation protocol. This protocol largely inhibited the nociception induced by PNV (3 µg/paw) (80±2%) (Figure 9A), and the nociception caused by the selective TRPV1 agonist capsaicin (1 nmol/paw) was abolished (Table S1). The sensory fibres that express TRPV1 also produce and release tachykinin after their depolarisation, an effect that could contribute to the development of nociception [40]. Interestingly, we observed that selective antagonists of the tackykinin NK_1_ (SR143033, 0.2 nmol/paw) or NK_2_ (SR48968, 20 nmol/paw) receptors were able to partially reduce PNV-trigged nociception with inhibitions of 57±10% and 40±8%, respectively (Figure 9B). Curiously, the combination of the NK_1_ and NK_2_ receptor antagonists was only as efficacious as the individual antagonists in reducing PNV-induced nociception (time of nociception of 124±4 s and 75±11 s for vehicle and SR143033 plus SR48968 groups, respectively; inhibition 40±9%).

These data suggest that PNV stimulates TRPV1 receptors and releases tachykinin in sensory fibres to induce a nociceptive effect.

## Discussion

The *Phoneutria nigriventer* sting is the most important form of envenomation by spiders in Brazil. Intense local pain is the main clinical manifestation of this spider sting, and the mechanisms involved in pain manifestation are not well described in the literature. In the present study, we studied the mechanisms through which PNV produces spontaneous nociception in mice using different pharmacological tools through blinded behavioural assessment with respect to drug administration. Our results show that an intraplantar injection of PNV venom in mice produced extended nociception that was mediated by the activation of sensory fibres through B_2_, TRPV1, 5-HT_4_ or ASIC receptor activation and the stimulation of tetrodotoxin-sensitive voltage-gated Na^+^ channels and kallikrein-like activity.

In our study, the PNV injection induced nociception that developed immediately and lasted for 4 hours. Our studies show that the dose that produced nociception in mice (3 µg/paw or 85.7 µg/kg) was close to the dose reported by [41] as the amount of *P. nigriventer* venom release (2.6–61.7 µg/kg per spider in an adult human being). The inoculation of *P. nigriventer* venom in humans and dogs promotes a local, spontaneous, unbearable pain that develops quickly after the sting and can last for many hours [1], [18], [42]. At first, we only recorded the amount of time spent licking the injected paw as the single index of pain because it is the most frequent parameter used to measure on-going pain in mouse models, as in the formalin, capsaicin and bradykinin tests [11], [33], [14]. Using this parameter, we detected a short nociceptive effect induced by PNV, as opposed to the long-lasting pain observed in humans and dogs. It is not surprising that the task of recognizing pain in rodents is difficult, given their ecological niche as prey animals [43]. Therefore, it has been reported that there are significant advantages to using a combination of several behaviours rather than a single index of pain in terms of sensitivity, specificity and duration [14], [15]. When other pain-related behaviours in addition to paw licking (such as paw lifting and favouring) were recorded, we were able to detect long-lasting (up to 4 hours) ongoing nociception. Furthermore, we observed that PNV injection produced paw oedema with a time course similar to the pain-related behaviours. Our data are in accordance with a previous study in which the peripheral injection of PNV in rats evoked a long-lasting (up to 6 h) mechanical hyperalgesia and paw oedema [44]. Thus, the long-lasting nociception seems to be related to the inflammatory process induced by PNV. Moreover, the combination of several behaviours rather than a single index in mice may be useful to measure pain after PNV injection as it better mimicked PNV envenomation in humans, indicating that the mice model may help to manage/optimise acute pain control in humans.

There are no clinically validated therapies for treating PNV-induced pain. The treatment of pain in humans is typically symptomatic. Local anaesthetic infiltration is often used. If the pain persists, a specific serum therapy, analgesics or opiates may be used [2]. It was reported that the pain factor after a PNV injection in dogs may be neutralized by a specific antivenin [8], [18]. We detected that the arachnid antivenin produced an anti-nociceptive effect when it was co-administered with PNV in mice, but not when it was administered 55 min after PNV injection. These findings indicate that substances presented in PNV and neutralised by the antivenin are important to pain development, but not to pain maintenance, which seems to be dependent on the inflammatory process. In line with this idea, the non-steroidal anti-inflammatory drug indomethacin did not prevent nociception development when administered before PNV, but was able to partially reverse nociception (Figure 2) and oedema (data not shown) when administered 30 min after PNV. We also tested some other clinically used analgesics for the treatment of PNV-induced nociception. We observed that the local anaesthetic lidocaine and the opioid morphine are able to prevent, but not reverse, PNV-induced nociception in mice. Interestingly, PNV-induced nociception was both prevented and reversed by dipyrone and acetaminophen. In accordance with these results, dipyrone is widely used in Brazil to treat pain after spider bites when a local anaesthetic is not sufficient to eliminate pain [45]. Thus, our model could be useful for investigating not only the mechanisms responsible for the development and maintenance of PNV-induced pain but also effective therapies for treating pain related to *P. nigriventer* in humans.

We next focused on the substances and mechanisms involved in the development of PNV-induced pain. Previous studies have indicated that dialysed venom seems to be less painful in dogs [3], which suggests that the nociception may be due to the presence of low-molecular-weight substances in PNV. In fact, the pro-inflammatory mediators histamine, serotonin and glutamate are present in PNV [9], [46]. We were able to detect these mediators in PNV and observed that dialysis reduced the both the amount of these mediators and the nociceptive effect.

Glutamate induces nociception in mice via the peripheral activation of NMDA, AMPA and kainite receptors [47]. We found that neither NMDA nor AMPA/kainate antagonists were able to reduce PNV-induced nociception at doses that were able to reduce the glutamate-trigged nociceptive effect. A possible explanation for this finding is that the low amount of glutamate present in *Phoneutria nigriventer* venom (0.5 mg/g PNV or 0.00017 µmol/paw in 3 µg/paw of PNV) was approximately 60 thousand times lower than the concentration used to induce a nociceptive effect (10 µmol/paw).

Histamine derived from venom or mast cell degranulation has been implicated in several of the biological effects of animal venoms [48], [49]. In the present study, we found that mast cell depletion, which we previously demonstrated reduced the amount of tissue histamine by 88% [12], is ineffective for reducing the algesic effect produced by PNV. Based on the finding that the mast cell degranulator compound 48/80 (derived from bee venom) was capable of inducing nociception, it is concluded that mast cell mediators may release algogenic substances, but it is not the mechanism responsible for PNV-induced nociception. Although we found that PNV contains histamine, an H_1_ receptor antagonist was not able to reduce the PNV-induced nociception. Of note, several studies have shown that histamine is important for the induction of oedema and pruritus, but not nociception [50]. In accordance with these findings, we observed that histamine induced oedema but not nociception when injected into the mice paw. Thus, neither the histamine derived from venom nor mast cell degranulation is related to the nociceptive effect of PNV.

The stimulation of serotonin receptors seems to be relevant to the production of pain [51]. Although we found serotonin in PNV, the amount (0.23 µg/ml or 0.23 mg/g PNV or 4 pmol/paw in 3 µg/paw of PNV) was approximately 25 thousand times lower than the concentration needed to induce a nociceptive effect in mice (100 nmol/paw), suggesting that serotonin is not relevant to the PNV-induced nociception. Moreover, we observed that serotonin caused nociception primarily through the 5-HT_3_ and not the 5-HT_4_ receptors, based on our finding that the co-administration of GR113908 did not reduce the nociceptive effect induced by serotonin. In contrast, PNV-trigged nociception is mediated by 5-HT_4_ but not 5-HT_3_ receptors. Our findings are in agreement with the electrophysiological study conducted by Costa and collaborators (2003) [52], which found that PNV induced rat vagus nerve depolarisation via the 5-HT_4_ but not the 5-HT_3_ receptor. Because this study used dialysed PNV, this effect does not appear to be mediated by a low-molecular-weight substance such as serotonin. Thus, PNV-induced nociception is dependent on 5-HT_4_ stimulation by a still unknown and most likely high-molecular-weight molecule. However, more studies on this topic are needed.

It was expected that voltage-gated Na^+^ channels would contribute to PNV-induced nociception, given that local anaesthetics reduce PNV-induced pain in humans [2] and nociception in mice (current study). Furthermore, we observed that TTX was very efficacious in reducing PNV-induced nociception in mice without altering the nociception induced by PGE_2_, indicating an important role for tetrodotoxin-sensitive voltage-gated sodium channels in the pain caused by PNV, but not by PGE_2_. This is in agreement with a report that PGE_2_-induced nociception is mediated by tetrodotoxin-resistant sodium channels in rodents [53]. Moreover, our findings suggest that the inhibitory effect of TTX in PNV-induced nociception was not simply due to conduction block in sensory neurons. Of note, families of small (3–9 kDa) peptide toxins have been detected in the venom of *P. nigriventer* [51]. Fraction 2 of PNV venom (PhTx2) contains at least two peptide toxins, namely PnTx2-5 and PnTx2-6, that directly bound and decreased the inactivation of tetrodotoxin-sensitive Na^+^ channels and facilitated their activation, thus inducing membrane depolarisation in vitro [54], [55], [56], [57]. However, more studies are needed to elucidate whether PNV components directly or indirectly activate voltage-sensitive sodium channels to induce nociception in vivo.

In addition to peptide toxins, some larger (>10 kDa) protein components of PNV have also been described [54]. Our results also show that boiling PNV to induce protein denaturation reduced the nociceptive effect. Moreover, the PNV dialysed in membrane with a MWCO of 12–14 KDa presented a lower, but still significant, nociceptive effect. Thus, we suggest that both low- and high-molecular-weight components contribute to PNV-induced nociception. The presence of proteases in the PNV has been previously demonstrated *in vitro* [54], and another report showed that PNV contains a non-dialysable component with tissue kallikrein-like activity *in vivo* that is involved in venom-induced oedema in rabbits [5]. Thus, we tested the possible involvement of the kallikrein-kinin system in PNV-induced nociception using biochemical and pharmacological tools. First, we detected a potent kallikrein-like activity in the venom *in vitro* that was active against a synthetic substrate and cleaved a low-molecular-weight kininogen to generate kinins. Second, based on the substrate preference (low- versus high-molecular-weight kininogen) and the sensitivity to inhibitors (aprotinin, but not SBTI) [58], [25], we suggest that this activity resembles a tissue kallikrein-like enzyme. Third, we observed that inactivating (by boiling) or inhibiting the kallikrein-like activity reduced PNV-trigged nociception. Consistent with our data, the PNV-induced oedema formation in rabbit skin involves local kinin synthesis in response to tissue (but not plasma) kallikrein-kininogen-kinin system activation [6]. Thus, the kallikrein-like activity plays a critical role in PNV-induced nociception, but further studies are needed to identify this component. Given that the tissue kallikrein of PNV cleaves kininogen, it could promote the generation of kinins and the stimulation of B_2_ receptors present in sensory fibres to induce nociceptor depolarisation and a consequent pain response [36]. In fact, we found that the tissue protease inhibitor aprotinin and the B_2_ receptor antagonist HOE 140 inhibited PNV-induced nociception. B2 receptor antagonists prevented PNV-induced plasma extravasation in rabbits [6] and nociception in mice (current study), but not plasma extravasation in rats [7]. This discrepancy may be related to the inter-species variability of the kinin system [59] and by the 10- to 100-fold lower dose of PNV (and consequently low kallikrein-like activity) used in the rat study (0.3 µg/site) compared to the rabbit (30 µg/site) or mice (3 µg/paw) studies.

It is well known that kinin activity at B_2_ receptors excites sensory nerve endings by both the activation and sensitisation of TRPV1 receptors *in vivo* [11]. We observed that the antagonism of TRPV1 or the ablation of TRPV1-positive sensory fibres reduced the PNV-induced nociception. In addition to the indirect effects, it has been recently demonstrated that spider venoms may contain toxins that directly bind to and stimulate the TRPV1 receptor [37], [38], [39]. Accordingly, we observed that low concentrations of PNV are capable of activating HEK cells expressing TRPV1 receptors, but not control cells. Moreover, our binding assay demonstrated that PNV components do not bind to the TRPV1 receptor at the intracellular vanilloid site, similar to other spider venom toxins that interact at extracellular sites of TRPV1 [37], [38], [39]. However, the exact components presented in PNV that stimulate TRPV1 and their site of interaction must be further identified. Our data indicate that, besides indirect mechanisms, PNV may contain components that directly bind and stimulate the TRPV1 receptor.

It has been well established that the treatment of animals with TRPV1 receptor agonists, such as RTX or capsaicin, produces a selective ablation of TRPV1-positive sensory fibres and largely reduced the amount of the neuropetides substance P and neurokinin A [11], [60], [61], [62]. We observed that the ablation of such peptidergic fibres significantly inhibited PNV-induced nociception. The reduced time that the animals spent licking the paws could theoretically be caused by motor impairments or sedation induced by the treatment RTX, rather than reflecting an analgesic effect. However, the number of crossings in the open-field test and the per cent of sedation in the platform test were similar in vehicle and RTX groups (data not shown), excluding this possibility.

In addition to the ablation of peptidergic fibres, the antagonism of the tachykinin NK_1_ and NK_2_ receptors also significantly inhibited PNV-induced nociception. Consistent with this, it was reported that peripheral tachykinin receptor antagonists reduced PNV-induced nociception in rats [44]. TRPV1-positive sensory fibres express NK_1_ and NK_2_ receptors as well as produce the selective ligands substance P and neurokinin A, which are released in response to nociceptive stimulation and may produce paracrine or autocrine effects that amplify the nociceptive process [63]. In fact, NK_1_ and NK_2_ receptors expressed by sensory nerves cause sensitisation of TRPV1 receptors, increase neuronal excitability and induce nociception in rodents [63], [64], [65]. We observed that a combination of NK_1_ and NK_2_ receptor antagonists was as efficacious as the antagonists alone to reduce PNV-induced nociception, but was less efficacious than sensory nerve ablation. These data indicate that NK_1_ and NK_2_ receptors use a convergent pathway to induce nociception (such as an indirect stimulation of the TRPV1 receptor) and that other sensory nerve-dependent mechanisms (such as a direct stimulation of TRPV1) are related to PNV-induced nociception. Thus, the nociceptive stimulation produced by PNV seems to release neuropeptides from sensory fibres, which sensitise the TRPV1 receptor by acting on NK_1_ and at NK_2_, and might amplify the nociceptive response. Of note, though NK1 receptor antagonists are usually able to inhibit pain in sensitised states and thus reverse hyperalgesia, we observed that tachykinin receptor antagonists prevented PNV-induced nociception. In accordance, subcutaneous injection of substance P has been shown to induce continuous pain in humans and nociception in rodents [64], [66], and peripheral NK1 and NK2 antagonists may prevent nociception induced by several algogens such as capsaicin and formalin [67].

Finally, we also studied the involvement of acid-sensing ion channels in PNV-induced nociception because these ion channels are an important target of several animal venoms [68]. Our results indicate that the ASIC receptor is involved in PNV-induced nociception because the co-administration of the ASIC blocker amiloride inhibited the PNV nociceptive effect. PNV could produce ASIC activation by both direct and indirect mechanisms. It has been demonstrated that the stimulation of Gq protein-coupled receptors, such as the B_2_ or 5-HT_4_ receptors that mediate PNV-induced nociception, may stimulate ASICs through protein kinase C [69]. Furthermore, it has been shown that arachidonic acid, in a manner independent of its metabolisation by cyclooxygenase, markedly sensitizes ASICs [70]. Accordingly, we observed that the cyclooxygenase inhibitor indomethacin did not reduce PNV-induced nociception. Of note, phospholipase A_2_, the enzyme responsible for arachidonic acid production, was detected in PNV [71]. Finally, we cannot exclude the possibility that PNV components could directly activate ASICs, as it was recently described that snake toxins isolated from *Micrurus tener* venom induce nociception through the direct stimulation of ASICs in sensory neurons [72]. However, more specific studies are needed to better understand this mechanism. Thus, PNV stimulates ASICs to produce nociception, an effect that could be mediated by both direct and indirect mechanisms.

In summary, the current results show that both the low- and high-molecular-weight substances in PNV produce spontaneous nociception action through direct or indirect activation of B_2_, TRPV1, 5-HT_4_ or ASIC receptors and the tetrodotoxin-sensitive voltage-gated Na^+^ channels present in primary afferents neurons, but not in mast cells. The elucidation of the mechanisms responsible for the nociception induced by PNV is of interest to better treat envenomation by *P. nigriventer* and understand the diversity of targets triggered by PNV toxins.

## Supporting Information

Table S1 **Controls for the pharmacological treatments.** AA = arachidonicacid ; Indo = Indomethacin; Glut = Glutamate; 5-HT = Serotonin; Methy = methysergide; Ondan = ondansetron; Resinf = resinferatoxin; C-48/80 = compound 48/80 Data are presented as the mean±SEM **P<0.01, ***P<0.001 compared with the vehicle group, #P<0.05, ##P<0.01, ###P<0.001 compared with the nociceptive substances group; one-way analysis of variance followed by Student-Newman-Keuls test.
(DOC)
