# Supplementary material for: Mechanisms Involved in the Nociception Triggered by the Venom of the Armed Spider Phoneutria nigriventer
Source: PLoS Negl Trop Dis. 2013 Apr 25;7(4):e2198. doi: 10.1371/journal.pntd.0002198 (PMC3636088; doi:10.1371/journal.pntd.0002198)
Supplement: Table S1 — Controls for the pharmacological treatments. AA = arachidonicacid ; Indo = Indomethacin; Glut = Glutamate; 5-HT = Serotonin; Methy = methysergide; Ondan = ondansetron; Resinf = resinferatoxin; C-48/80 = compound 48/80 Data are presented as the mean±SEM **P<0.01, ***P<0.001 compared with the vehicle group, #P<0.05, ##P<0.01, ###P<0.001 compared with the nociceptive substances group; one-way analysis of variance followed by Student-Newman-Keuls test. (DOC) [file pntd.0002198.s001.doc]

**Table S1. Controls for the pharmacological treatments**

| **Algogenic agent/paw** | **Control group Nociception (s)** | **Treated group Nociception (s)** | **Treatment Scheme** |
| --- | --- | --- | --- |
| PBS/Vehicle | 4±3 | - | - |
| AA100 nmol | 58±5** | Indo30 µmol | 17±11^#^ |
| Glut 10 μmol | 58±4** | DNQX 10 nmol | 22±4^#^ |
| Glut 10 μmol | 67±9** | MK-801 10 nmol | 34±8^#^ |
| 5-HT 100 nmol | 52±4** | Methy10 nmol | 1±1^###^ |
| 5-HT 100 nmol | 57±11** | Ondan30 nmol | 9±4^##^ |
| 5-HT 100 nmol | 41±7** | GR113808 15 nmol | 42±10 |
| PGE_2_ 3 nmol | 58±12 | TTX 20 pmol | 54±14 |
| C-48/80 10 µg | 102±8** | C-48/80 10 µg | 10±3^###^ |
| Caps 1 nmol | 63±9*** | Resinf pre-treated | 0±0^###^ |
